# Biosynthesis and antibacterial activity of manganese oxide nanoparticles prepared by green tea extract

**DOI:** 10.1016/j.btre.2022.e00729

**Published:** 2022-04-11

**Authors:** Wahran M. Saod, Layth L. Hamid, Nisreen Jassam Alaallah, Asmiet Ramizy

**Affiliations:** a Chemistry department, College of science, University Of Anbar, Ramadi, Iraq; b Biology department, College of science, University Of Anbar, Ramadi, Iraq; c Department of Chemistry, Directorate of Education Anbar, Ministry Of Education, Iraq; d Physics department, College of science, University Of Anbar, Ramadi, Iraq

**Keywords:** Manganese oxide, Nanoparticles, Biosynthesis, Green tea extract, Antibacterial

## Abstract

Amongst chemical and physical techniques, the biosynthesis method of metal nanoparticles has received the interest of many researchers owing to its environmental safety, simplicity and inexpensiveness. Manganese oxide nanoparticles (MnO NPs) were successfully synthesised using green tea extract as the reducing agent and characterised by UV–Vis spectroscopy, X-ray diffractometry and Fourier transform infrared spectroscopy. The shape and size of the MnO NPs were obtained by scanning electron microscopy. The size of the MnO NPs was 20–30 nm. The MnO NPs exhibited strong antibacterial activity against pathogenic bacteria, namely, *Escherichia coli, Klebsiella pneumoniae* and *Pseudomonas aeruginosa*, with inhibition zones of 12, 14 and 18 mm, respectively. Moreover, the minimum inhibitory concentration (MIC) of the MnO NPs was 12.5 U/mL as determined by resazurin microtitre assay. The activities of some antibiotics remarkably increased when combined with MnO NPs (at MIC).

## Highlights

• Manganese oxide nanoparticle (MnO NPs) successfully synthesized and confirmed by many techniques.
• The characteristic nanoparticles properties of MnO NP were proved by UV–Vis, XRD and FTIR. The shape and size of MnO NPs were demonstrated by SEM equipment.
• MnO NP exhibited strong antibacterial activity when scanned against pathogenic bacteria.

## 1 Introduction

The biosynthesis of metal oxide nanoparticles (NPs) by plant extraction has achieved popularity in the last years owing to its eco-friendliness and pharmaceutical properties. This type of nanoparticle synthesis has benefits over chemical and physical methods because it has no risk on the environment; therefore, it can be used for biomedical purposes [1].

MnO has gathered the interest of many researchers owing to its influence and electromagnetic characteristic [2]. Various methods have been designed for the synthesis of MnO, such as self-reacting microemulsion [3], deposition [4] and solid reaction [5]. However, employing natural products to reduce and stabilise Mn metal into nanoparticles is more eco-friendly, inexpensive and easier in comparison with the methods above [6, 7]. Green tea is a well-known medicinal plant that has a broad healing activity owing to the presence of phenolic groups in its composition [8].

Pathogenic bacteria, such as *Escherichia coli, Klebsiella pneumoniae and  Pseudomonas aeruginosa*, can affect people with compromised natural defenses and cause extreme systematic disease [9]. Clinical manifestations include nosocomial or healthcare-related infections, such as pneumonia, urinary infections, surgical wound infections, joint infections and septicaemia, as well as common community infections, such as intestinal, skin, soft tissue and otitis externa infections [10].

Antibiotic resistance and biofilm formation are global public health problems that lead to high morbidity, mortality and healthcare costs [11, 12]. Metal oxide NPs have the potency to contend antibacterial resistance by many mechanisms. NPs can penetrate biofilms and bacterial cell walls and cause cytotoxic effect owing to their nanoscale size; they can increase the effectiveness of current antibiotics by protecting them from detection and supply a means of targeted delivery to microorganisms to maximise the topical concentration and bactericidal impact of the agent [13].

In this paper, we aimed to synthesise MnO NPs using green tea leaf extract as the reducing and capping agents, characterise the synthesised MnO NPs and estimate its antibacterial activity towards Gram-negative pathogenic bacteria, alone and in combination with other antibiotics.

## 2 Materials

Methanol, manganese sulfate (MnSO_4_) and sodium hydroxide (NaOH) were purchased from Sigma Aldrich, USA. MacConkey culture media, blood agar media and eosin–methylene blue agar were purchased from HiMedia (India).

## 3 Methods

### 3.1 Preparation of green tea extract

**Fig. 1:**
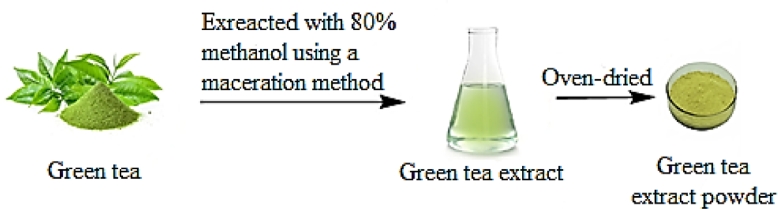
Green tea extract process.

Fresh green tea leaves were purchased from a local market in Ramadi, Al Anbar, Iraq. the leaves were cleaned, dried and ground, and 50 g green tea powder was extracted with 500 mL of 80% methanol by maceration method [14]. The extract was oven-dried at 40 °C and kept in densely locked vials as shown in Fig. 1.

### 3.2 Biosynthesis of MnO NPs

**Fig. 2:**
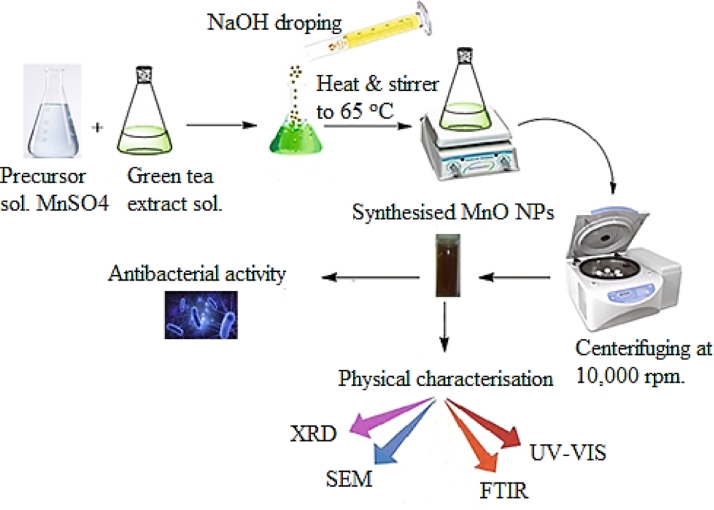
Preparation and characterisation of manganese oxide nanoparticles.

Green tea extract (10 mL, 100 µg/mL in distilled water) were mixed with 100 mL of 0.1 M MnSO_4_ stock solution. Drops of NaOH solution were added into the solution under gradual and constant stirring until the pH reached 8 to achieve tiny-sized particles [15]. The mixture was stirred with a magnetic stirrer at 65 °C for 6 h. The solution's colour changed from green to dark brown, which indicates the biosynthesis of MnO NPs. The mixture was centrifuged at 10,000 rpm for 10 min, and the supernatant was discarded. The pellet was washed 4–5times as shown in Fig. 2.

### 3.3 Characterisation of MnO NPs

The morphology (size and shape) of the MnO NPs was characterised by scanning electron microscopy (SEM). The structural properties of the MnO NPs were investigated by X-ray diffractometry (XRD) analysis (Philips PW1730). The optical properties of the MnO NPs were estimated using an UV–visible spectroscopy (Shimadzu UV-160A). FTIR spectroscopy (Shimadzu IRAffinity˗1) was utilised to examine the colloidal solution of MnO NPs.

### 3.4 Bacterial samples

Bacterial isolates from cases from Ramadi Teaching General Hospital were collected and grown on MacConkey culture media, blood agar media and eosin methylene blue agar and incubated at 37 °C for 18–24 h. The cultivation characteristics of the colonies were observed, and a Gram stain was performed to determine the isolates' Gram positivity and negativity. The isolates were biochemically tested for diagnosis, and all Gram-negative bacterial isolates were verified by VITEK 2 compact.

### 3.5 Antimicrobial susceptibility test

Well diffusion, disk diffusion and resazurin microtitre assay (REMA) were used to test the antibacterial activity and minimum inhibitory concentration (MIC) of the green tea MnO NPs. Firstly, the antimicrobial activity of the synthesised MnO NPs was observed against *E. coli*, *K. pneumoniae* and *P. aeruginosa* by well diffusion assay [16]. Then, the lowest concentration with no change in resazurin colour was considered the MIC of the MnO NPs [17]. Nine antibiotics (cefixime, ampicillin, augmentin, levofloxacin, ceftriaxone, gentamicin, trimethoprim, norflexin and doxycycline) were tested on Gram-negative bacteria. Bauer–Kirby assay was chosen to test the antibiotics alone and in combination with MnO NPs (Matuschek et al., 2014; CLSI, 2020). The inhibition zones around the wells and discs were measured (millimetre) and recorded after 18 h of overnight incubation at 37 °C [18].

## 4 Results and discussion

### 4.1 UV–Vis spectral analysis

**Fig. 3:**
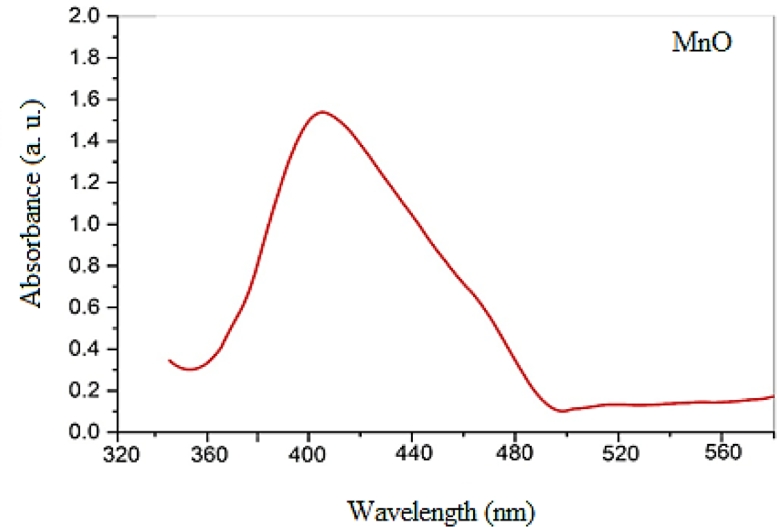
UV–Vis spectral result for prepared MnO nanoparticles.

The brown colour of the prepared nanocomposite was the result of the surface plasmon resonance (SPR) caused by the oscillation of electrons on the surface of the nanocomposite. The shade of the compound depends on two main factors, namely, the shape of the compound and the size of the NPs. The UV–Vis spectrum of the MnO NPs solution shows a peak in the visible region at 410 nm (Fig. 3). Theoretically, MnO NPs possess an absorption peak in the range of 350–410 nm.

### 4.2 FTIR analysis

**Fig. 4:**
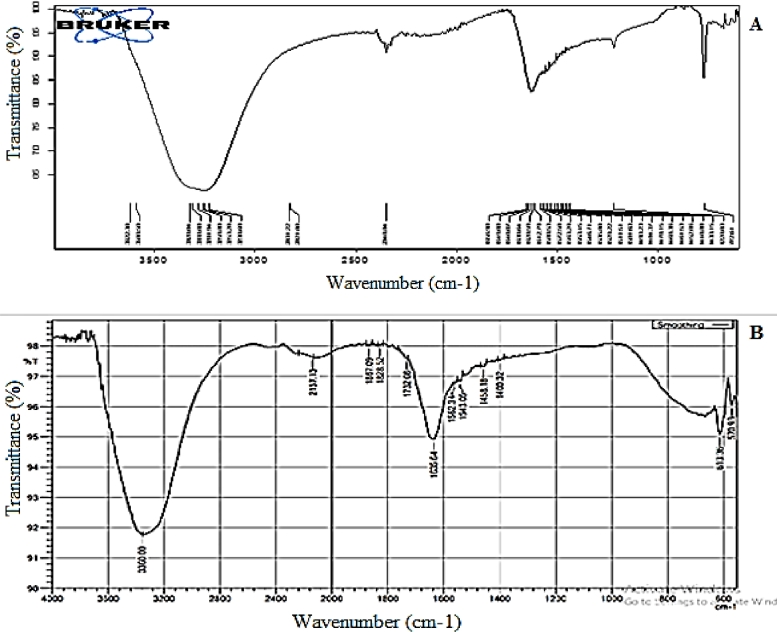
(A) FT-IR spectrum of the green tea extract, (B) FT-IR spectrum of the biosynthesised nanoparticles of MnO by green tea extract.

As shown in Figs. 4A and B, the functional groups in green tea extract were identified, and their role in MnO NP synthesis was analysed. The FTIR analysis results indicate the existence of bio-components on the NPs’ surface, such as alkaloids, tannins and glycosides. FTIR measurements were utilised to determine the interaction between manganese salts and protein molecules capable of reducing Mn ions and stabilising MnO NPs. FTIR analysis revealed some remarkable bands of the vibrations of the hydroxyl group, C–O, MnO and others. This corresponds to the MnO NPs and bio-compounds on the surface [19, 20].

As shown in Fig. 4B, two strong peaks at 613 and 570 cm^−1^ arise from the stretching vibrations of the Mn–O and Mn–O–Mn bonds [21]. In addition, the strong absorptions at 3360 cm^−1^ and the weak absorptions at 2800–3000 cm^−1^ indicate the stretching vibrations of O–H and C–H, respectively. These results are consistent with the C–OH stretching and OH bending vibrations; the bands at 1400, 1458, 1543, 1562 and 1635 cm^−1^ correspond to C–O and O–H [22, 23]. These results were acquired from the FTIR results. The results indicate that some organic residues, such as hydroxyl and carboxyl groups, are present on the surface of the prepared MnO NPs.

### 4.3 XRD analysis

**Fig. 5:**
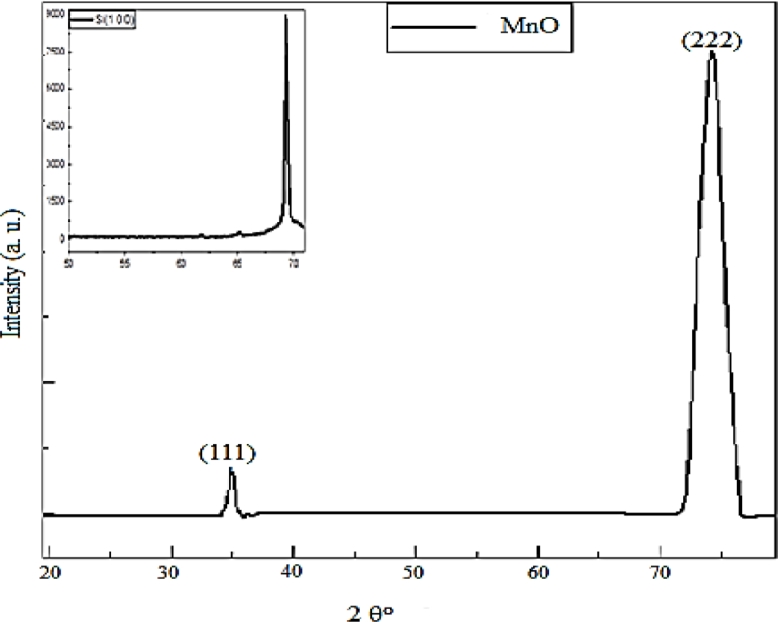
XRD patterns of MnO nanoparticles prepared by green tea extract.

Fig. 5. showed that XRD measurement of MnO Nanostructures deposition on silicon substrate. The diffraction peaks at 2*θ* around 35.079° and 73.952° can be readily indexed to (111) and (222) related to MnO thin film samples which indicated that our samples have cubic structure according to (PDF#07–0230) and these result in agreement with other researcher groups [24, 25],from other hands the peak located at2*θ* of 69.5° was below the silicon substrate with orientation (1 0 0). Moreover, the expansion of the diffraction peaks is an indication that the prepared material is in the nanometre range The average crystallite size of the deposited thin films is calculated to be 14.9 nm from the line broadening using Scherrer formula below [26]

(1) D=Kλ/βcosΘ

Where λ represents the wavelength of the x-ray radiation 0.154 nm, βhkl Full width half of the maximum diffraction peak (radians), θ is the Bragg angle and (k) is form factor equal to 0.9.

### 4.4 SEM analysis

**Fig. 6:**
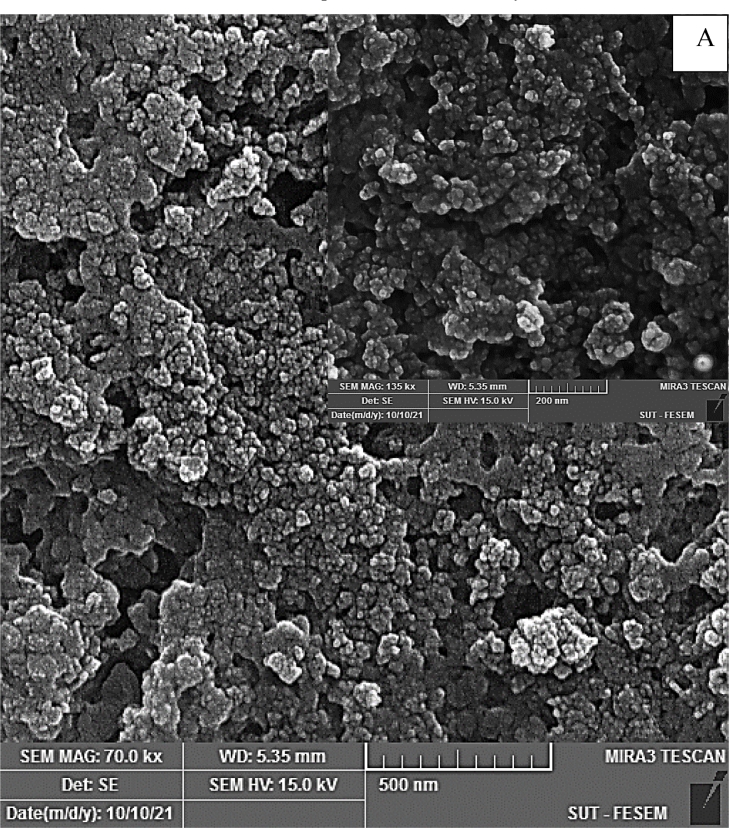

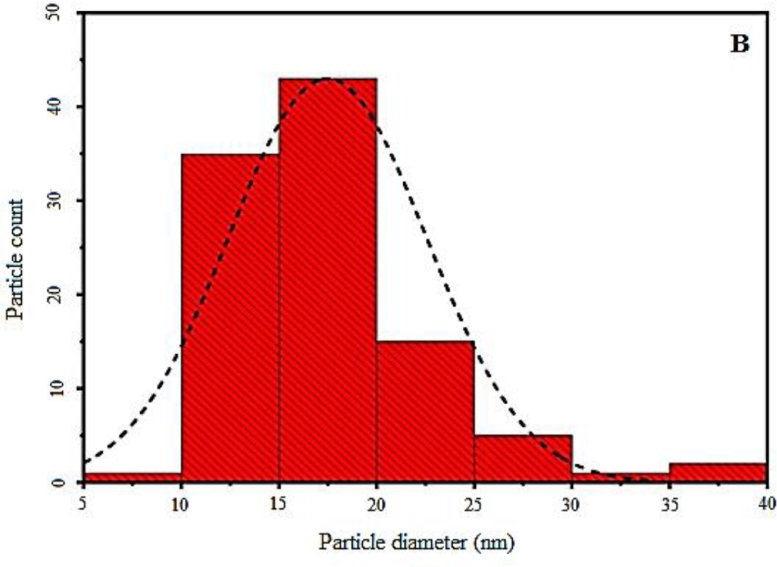
(A) FE-SEM image of MnO nanoparticles, (B) Size distributions of MnO nanoparticles.

Fig. 6A. showed the morphology of the Mno thin film with regularly distribution like-nano algae with high spread of atoms on the substrate, and this may be led to disappear of defect from the surface, on other hand, this may be due to the number of deposited atoms increases and thus increases of coulomb repulsion force which may be utilized for antibacterial activity toward gram-negative pathogenic bacteria alone and in combination with many antibiotics which will see later. There are smaller size distributions around 18 nm as shown in Fig. 6B. with random orientation were covered all the substrate.

### 4.5 Bacterial strains

In the current study, the antiobitic sensitivity of 12, 16 and 9 clinical isolates of *E. coli, K. pneumoniae* and *P. aeruginosa*, respectively, was tested. Multidrug-resistant isolates from each species were selected for antimicrobial susceptibility tests with and without MnO NPs. The present study evaluated the antimicrobial activity of MnO NPs towards *E.* coli, *K. pneumoniae* and *P. aeruginosa* alone and in combination with nine antibiotics.

### 4.6 Antibacterial activity of green tea MnO NPs

In the last years, intrinsic and acquired antibiotic resistance have hindered the treatment of infections and are therefore associated with substantial morbidity and mortality. With the rise in bacterial resistance to antibiotics, the synthesis of other classes of materials that can be combined with existing antibiotics for infection control has gained substantial interest [27].

**Fig. 7:**
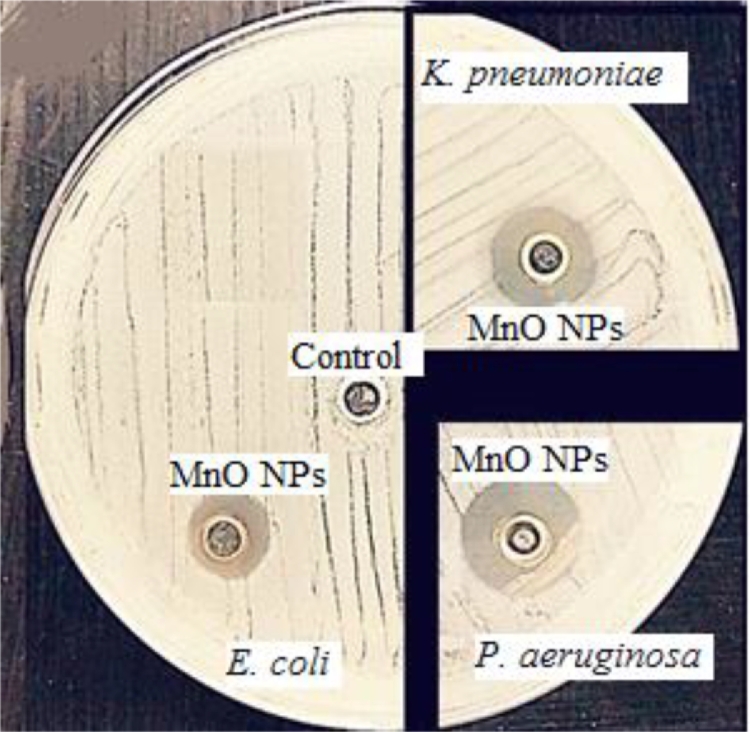
Scanned of antimicrobial activity of MnO NPs against E. coli, K. pneumoniae, and P. aeruginosa.

In our study, the antibacterial activity of MnO NPs alone or in combination with nine antibiotics against *E.* *coli, K. pneumonia*e and *P. aeruginosa* was tested by well diffusion and Bauer–Kirby methods. The MnO NPs showed vigorous activity towards *E. coli*, *K. pneumoniae*, and *P. aeruginosa* with inhibition zones of 12, 14 and 18 mm, respectively (Fig. 7). The REMA results showed that the MIC of the MnO NPs for the selected isolates was 12.5 U/mL. Many studies indicated that metal oxide NPs have achieved substantial attention because their antibacterial activity is based on their small size, which gives them the ability to penetrate bacteria and cause damage and toxicity to intracellular parts [16, [28], [29], [30], [31]].

**Fig. 8:**
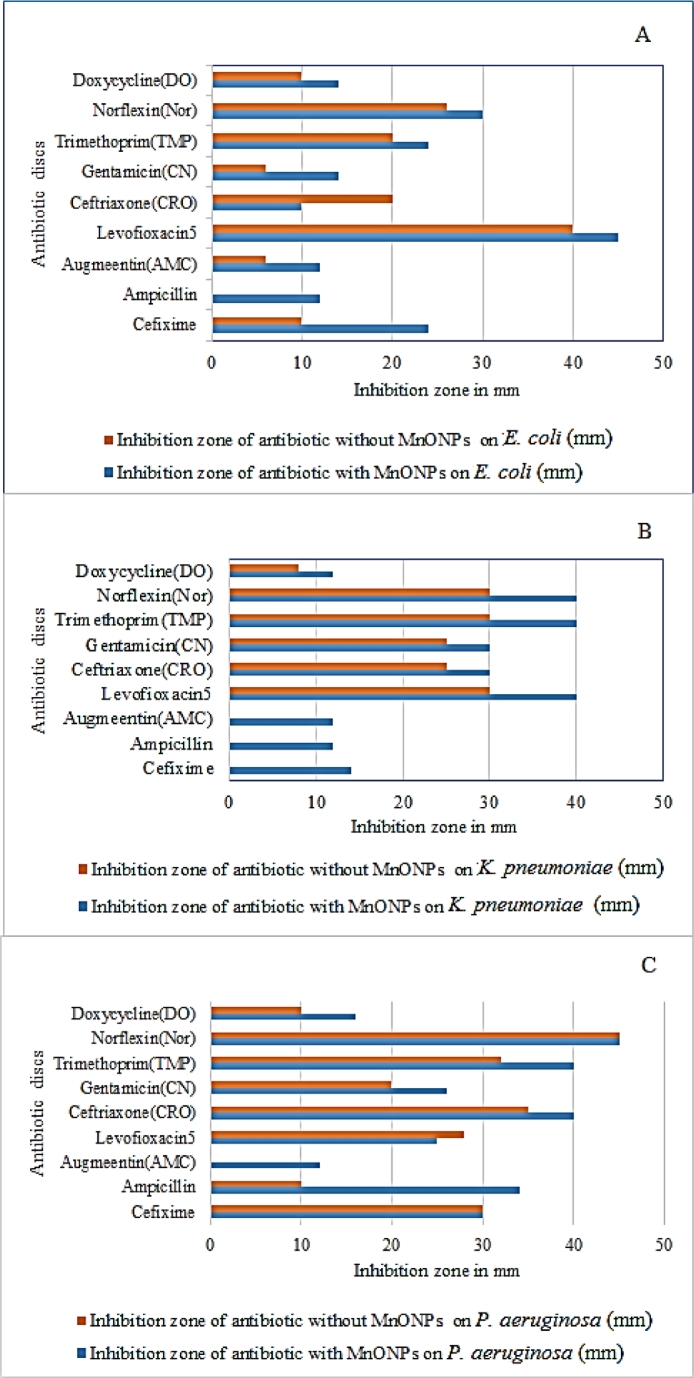
Antibacterial inhibition zones (millimetre) of antibiotics alone and in combination with MnO NPs on (A) E. coli, (B) K. pneumoniae, and (C) P. aeruginosa.

The antibiotic discs treated with MnO NPs (in MIC) showed a remarkable increase in inhibition zone compared with the antibiotic discs that were not treated with MnO MPs (Fig. 8). The synergistic effect between the antibiotics and MnO NPs is due to the nanosize of the particles and the large surface area; therefore, the antibiotic materials were easily incorporated and delivered inside the cells, distributed into transfer channels and cell walls and released metabolites more easily [30, 32].

## 5 Conclusion

MnO NPs were successfully synthesised by biosynthesis method using green tea extract as the stabilising and reducing agents. The synthesised MnO NPs were characterised using multiple techniques, which proved that the MnO NPs synthesised from aqueous green tea extract had bioactive groups. The MnO NPs synthesised by green tea inhibited the development of *E. coli*, *K. pneumoniae* and *P. aeruginosa*. Combining the MnO NPs with antibiotics increase the efficiency of the antibiotics against Gram-negative bacteria. MnO biosynthesis maybe deem as promising for the evolution of recent antimicrobials for pharmaceutical industry applications.

## Conflicts of Interest Statement

The authors whose names are listed immediately below certify that they have NO affiliations with or involvement in any organization or entity with any financial interest (such as honoraria; educational grants; participation in speakers’ bureaus; membership, employment, consultancies, stock ownership, or other equity interest; and expert testimony or patent-licensing arrangements), or non-financial interest (such as personal or professional relationships, affiliations, knowledge or beliefs) in the subject matter or materials discussed in this manuscript.
